# Effects of information-knowledge-attitude-practice health education combined with cluster-based care in patients with gestational hypertension

**DOI:** 10.1097/MD.0000000000035346

**Published:** 2023-10-13

**Authors:** Jiao Wen, Xiuping Liu

**Affiliations:** a Department of Obstetrics Nursing, West China Second University Hospital, Sichuan University, Key Laboratory of Birth Defects and Related Diseases of Women and Children Sichuan University, Ministry of Education, Chengdu, Sichuan, People’s Republic of China.

**Keywords:** cluster based care, hypertension during pregnancy, IKAP health education, postpartum depression, pregnancy outcome, prenatal anxiety, quality of life

## Abstract

To analyze the impact of information-knowledge-attitude-practice (IKAP) health education incorporated with cluster-based care on blood pressure control, pregnancy outcome and life quality in those who have gestational hypertension, and to provide methodological backing for the care of patients. A total of 80 patients with pregnancy-induced hypertension were selected as the research objects and randomly divided into control and experimental groups, with 40 cases in each group. The control group received routine cluster care, which included providing individual and group health information through the distribution of health education manuals to hypertensive patients during pregnancy and their families. The experimental group received additional IKAP health education, including data collection, health knowledge imparted, concept change and behavior generation process. Blood pressure control status was measured and recorded. Adverse pregnancy outcomes include placenta previa, cesarean section, hyperamniotic fluid, fetal distress, and postpartum hemorrhage. Postpartum quality of life conditions, including physical activity, emotional awareness, physical discomfort, mental health, sleep quality, postpartum anxiety or depression, and general health were evaluated. Age, prepregnancy BMI, and educational level did not significantly vary between the 2 groups(*P* > .05). In comparison to the control group, the experimental group demonstrated healthier behavior. Blood pressure and weight control during pregnancy were better than control group. The frequency of adverse pregnancy was inferior to control group. The number of adverse neonatal conditions was inferior to control group. The postpartum quality of life score was higher in the experimental group compared to the control group. The combination of IKAP health education and cluster based care has a better effect on blood pressure control compared to the sole use of cluster based care. This approach can reduce the likelihood of experiencing adverse pregnancy outcomes and help improve the quality of life for patients after delivery.

## 1. Introduction

Gestational hypertension is a common issue during pregnancy, and its clinical manifestations can vary depending on when it occurs.^[1,2]^ White coat hypertension, hidden hypertension and transient hypertension have been included in the diagnosis and treatment of hypertensive diseases during pregnancy in the expert consensus of 2019, indicating that the concept of early prevention, early detection and early treatment is particularly important for hypertensive diseases during pregnancy.^[3]^

In China, there has been a trend of women delaying their childbearing age, resulting in an increasing proportion of elderly pregnant women and greater attention on hypertensive diseases during pregnancy.^[4]^ The exact pathogenesis of hypertensive diseases in pregnancy is still not fully understood. The pathogenic causes and mechanisms of preeclampsia are very complex and are considered to be a disease with multiple factors, mechanisms and pathways.^[5]^ It has been theorized that when the infiltration ability of extravillus trophoblast cells is impaired, the recast ability of uterine spiral arteriole is inhibited, and placental implantation contribute to the occurrence of diseases.^[6]^

Hypertensive diseases during pregnancy can lead to detrimental effects on vital organs such as the cardiovascular, liver, kidney and brain organs. Related studies have demonstrated that placental function is inhibited due to insufficient placental injection in hypertensive patients during pregnancy. Fetal growth is limited, increases the likelihood of premature birth and low birth weight, and results in abnormal expression of substances like endothelin in umbilical cord blood.; Furthermore, these conditions affect the coagulation and fibrinolytic system, which limits the growth and development of the fetus, further increases the probability of neonatal death, and significantly deteriorates both adults and children’s physical and mental health.^[7]^ Related animal experiments have revealed that the newborns with fetal growth restriction exhibit decreased diaphragmatic contraction capacity, which leads to impaired respiratory function. In severe cases, it may affect the development of the nervous system, resulting in cerebral palsy, slow intellectual development and decreased control. The increased risk of metabolic diseases such as obesity and diabetes in childhood and adulthood also increases the financial and mental burden on their families.^[8]^

Studies have also shown a higher incidence of anxiety and depression in hypertensive patients during pregnancy compared to ordinary pregnant women; Poor mental state will affect the health of both mother and child, and postpartum anxiety and depression will affect their postpartum quality of life.^[9]^ With the improvement in living standards and advancements in medical and health technology, anxiety and depression during pregnancy have attracted the attention of scholars and experts.^[10]^ Studies have shown that prolonged anxiety during pregnancy can lead to small gestational age; The proportion of low fetal weight caused by negative emotions during pregnancy increased by at least 25% compared with normal pregnant women; In severe cases, it can also raise the probability of preterm birth and other adverse pregnancy outcomes. This highlights the importance of prenatal and postnatal care for hypertension during pregnancy.^[11]^ A separate study revealed that during the latter stages of pregnancy, there exists an inverse relationship between the severity of depression and the duration of walking as a physical activity. Additionally, walking was found to have a positive correlation with anxiety levels. Exercise holds promise as a potentially efficacious approach for addressing certain manifestations of depression.^[29]^ Engaging in appropriate physical activity during pregnancy not only yields psychological benefits for expectant mothers and enhances mood, but also exerts a positive impact on the regulation of fasting and postprandial blood sugar levels, as well as HbAc1, in pregnant women diagnosed with gestational diabetes. There exists a favorable correlation between the performance of physical activity and the management of gestational diabetes mellitus. Considering these factors, the study proposed an analysis of the impact of information-knowledge-attitude-practice (IKAP) health education combined with cluster based care on blood pressure control, pregnancy outcome and quality of life of pregnant patients with high blood pressure, hoping to improve suffers disease control and pregnancy outcome, as well as improve their postpartum quality of life.

## 2. Methods

### 2.1. Research object

The study used convenience sampling method to select 80 patients with pregnancy-induced hypertension who were hospitalized in West China Second Hospital of Sichuan University from April 2022 to April 2023. Patient examination indicators and data information are complete and reliable, and ethical standards should be strictly complied with in the selection process. The study was approved by the Ethics Committee of the Second Affiliated Hospital of Sichuan University (2021 Medical scientific rescarch for ethical approval No. 62).^[12]^ The following were the inclusion requirements: Patients were older than 18 years and younger than 35 years; Suffering from pregnancy-induced hypertension; The pelvic assessment is normal, the fetus is healthy and weighs more than 4 kg; Clear mental awareness, able to communicate and communicate normally; Single pregnancy; No bad habits such as smoking and drinking; Accept the experiment’s risks and complete the informed consent form. The following are the exclusion requirements: Hypertensive disease before pregnancy; Twin pregnancy or cannot be delivered vaginally; Have other medical and surgical complications or mental illness; Patients with a family history of hypertensive diseases during pregnancy; Gestational age >42 weeks; Can not visit at any time, cannot determine the specific person. The 80 participants who satisfied the requirements were split into 2 groups at random, with 40 patients in each of the control and experimental groups.

### 2.2. Research methods

In this study, 80 participants were divided into 2 groups, 40 of which were control group (CG), and routine cluster-based care was implemented. That is, individual and collective health promotion is carried out through the distribution of health education manuals to hypertensive patients during pregnancy and their families.^[13]^ 40 patients in the experimental group were combined with IKAP health education on the basis of the control group. The specific measures were as follows; Data collection: Collect all kinds of information at admission, such as age, education level, living habits, eating habits and occupation; The researchers also gained a comprehensive understanding of the participants family situations and mental health status. Focus on the understanding of nutrition and hypertensive diseases during pregnancy; The demand of health education during pregnancy was analyzed and the corresponding education plan was made; Health knowledge teaching: Health education for hypertensive patients in pregnancy was conducted through effective communication skills; The language of the nursing staff responsible for health education should be easy to understand and speak at a moderate speed to avoid the boredom of patients; At the same time, it is necessary to observe the reaction of patients and their families to ensure that they fully understand the content of health education. The specific teaching contents include rationalizing diet, making a corresponding diet plan according to the patient’s gestational age and blood pressure control, and teaching the patient to flexibly adjust the diet plan using the food exchange method; Actively communicate with patients, provide them with psychological counseling, improve treatment confidence; The patient’s blood pressure self-test, as far as possible to ensure that the blood pressure is within a reasonable and controllable range; Patients need to carry out reasonable health exercise during pregnancy, aerobic exercise under the guidance of medical staff, and run for about half an hour after meals; Change of concept: change the health knowledge and cognition of patients and their families, understand the impact of bad habits on health, and ensure that they maintain good living habits; Behavior generation: After receiving health knowledge education and guidance, patients self-concept changed, leading them to consciously implement healthy behavior activities, and ensure the health of mothers and infants in accordance with health education plans.

### 2.3. Observation indicators

The general information of the 2 groups included age, prepregnancy BMI, and education level. Healthy behaviors mainly include diet control, proper exercise and regular blood pressure measurement; Pregnancy indicators include amniotic fluid index, BMI, pregnancy weight gain, and blood pressure measurements. Pregnancy outcomes included placenta previa, cesarean section, hyperamniotic fluid, fetal distress, and postpartum hemorrhage. Neonatal conditions were divided into neonatal asphyxia, neonatal pneumonia, neonatal hypoxia, neonatal growth retardation and hyperbilirubinemia. The labor pain of the 2 groups was compared by digital pain scoring method. The anxiety degree of patients was quantified by referring to the clinical anxiety score scale. Nursing satisfaction includes 3 kinds: very satisfied, satisfied and dissatisfied, and very satisfied and satisfied are total nursing satisfaction. Postpartum quality of life conditions include physical activity, emotional awareness, physical discomfort, mental health, sleep quality, postpartum anxiety or depression, and general health.^[14]^

### 2.4. Statistical methods

The research data were analyzed by SPSS 25.0 software. Counting data were tested by chi-square distribution. The measurement data adopts the standard of mean plus minus. *t* test, variance test and rank test were used for comparison between groups.^[15]^

## 3. Results

### 3.1. Background data for the 2 patient cohorts

In this study, 80 participants who met the requirements were allocated into 2 groups at random, 40 patients in each CG and For example, General information, including age, prepregnancy BIM and education level, was provided for both groups. Table 1 compares the exchange of generic data between the 2 parties.

**Table 1:** Comparison of general information between two groups of patients.

Baseline information	CG	e.g.,	*x^2^*	*P* value
	(n = 40)	(n = 40)		
Age(years)	29.88 ± 3.12	29.81 ± 3.03	0.04	.981
prepregnancy BMI (kg/m^2^)	21.04 ± 2.52	22.62 ± 2.82	3.642	.161
Education level	/	/	2.128	.344
High school or below	18.40%	15.30%	/	/
College degree or above	81.60%	84.70%	/	/

CG = control group.

In Table 1, the mean years of CG and For example, were 29.88 ± 3.12 years and 29.81 ± 3.03 years, respectively. prepregnancy BMI was 21.04 ± 2.52 kg/m^2^ and 22.62 ± 2.82 kg/m^2^, respectively. In the CG, the proportion of patients with high school education or below was 18.4%; The proportion of patients with college education or above was 81.4%; In the CG, the proportion of patients with high school education or below was 15.3%; The proportion of patients with college education or above was 84.7%. Age, prepregnancy BMI, and educational attainment were significantly different between the 2 groups.

### 3.2. Comparison of health behaviors between the 2 groups

**Figure 1.**
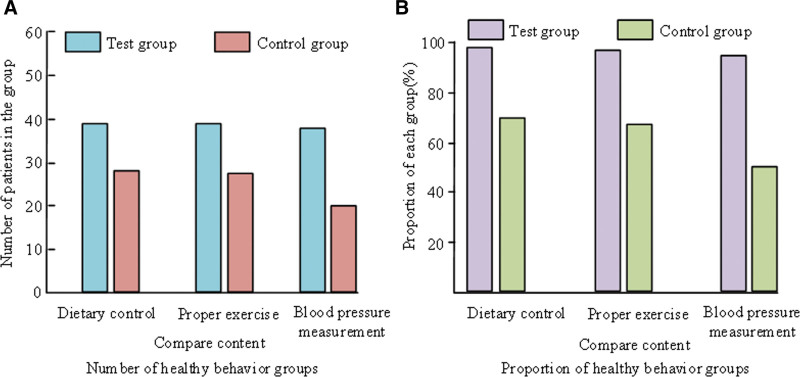
Comparison of health behaviors between two groups of patients.

The health behaviors of the patients in the study mainly included diet control, appropriate exercise and regular blood pressure measurement. The comparison of the health behaviors of the 2 groups of patients is shown in Figure 1.

In Figure 1, in the For example, and the CG, there were 39 and 28 instances, respectively, of diet control cases. The number of appropriate exercise cases was 39 and 27, respectively. The number of regular blood pressure measurement cases was 38 and 20, respectively. The diet control ratio of For example, and CG was 97.5% and 70%, respectively. The proportion of appropriate exercise was 97.5% and 67.5% respectively. The proportion of regular blood pressure measurement was 95% and 50% respectively. The rates of diet control, moderate exercise and regular blood pressure measurement in the For example, were increased by 27.5%, 30%, and 45%, respectively, compared with the CG. The 2 groups health-related behaviors differed significantly.

### 3.3. Comparison of pregnancy indexes between the 2 groups

Pregnancy indicators included amniotic fluid index, BMI, weight gain during pregnancy, and blood pressure measurements. Table 2 shows the indicators of pregnancy.

**Table 2:** Comparison of pregnancy indicators between two groups of patients.

Indicator name	CG	e.g.	*t*/*x^2^*	*P* value
	(n = 40)	(n = 40)		
Amniotic fluid index (mm)	125.81 ± 30.21	109.83 ± 28.72	2.434	.017
BMI (kg/m^2^)	21.64 ± 1.82	20.51 ± 1.62	3.021	.002
Weight gain during pregnancy (kg)	17.81 ± 2.54	16.59 ± 2.84	2.072	.041
Blood pressure measurement value (mm Hg)	135.12 ± 6.25	130.34 ± 5.32	4.856	<.001

CG = control group.

In Table 2, the measured values of amniotic fluid index, BMI, pregnancy weight gain and blood pressure in the CG were 125.81 ± 30.21 mm, 21.64 ± 1.82 kg/m^2^, 17.81 ± 2.54 kg and 135.12 ± 6.25 mm Hg, respectively. The measurements of amniotic fluid index, BMI, pregnancy weight gain, and blood pressure were 109.83 ± 28.72 mm, 20.51 ± 1.62 kg/m^2^, 16.59 ± 2.84 kg and 130.34 ± 5.32 mm Hg, respectively. The mean measurements of amniotic fluid index, BMI, pregnancy weight gain, and hypertension and high blood pressure were reduced by 15.98 mm Hg, 1.13 kg/m2, 1.22 kg/m2, and 4.78 mm Hg, respectively. Amniotic fluid index, BMI, weight gain during pregnancy, blood pressure and hypertension between the 2 groups varied significantly across pregnancies (*P* < .05).

### 3.4. Comparison of pregnancy outcomes between the 2 groups

Pregnancy outcomes included placenta previa, cesarean section, hyperamniotic fluid, fetal distress, and postpartum hemorrhage. Table 3 displays a comparison of the 2 groups pregnancy markers.

**Table 3:** Comparison of pregnancy indicators between two groups of patients.

Group	N	Placenta	Cesarean section	Polyhydramnios	Fetal distress	Postpartum hemorrhage
CG	40	3	20	12	10	12
e.g.,	40	1	10	2	2	2
	/	0.211	5.185	6.572	7.371	10.181
*P* value	/	.645	.023	.011	.007	.001

CG = control group.

In Table 3, the numbers of placenta previa, cesarean section, hyperamniotic fluid, fetal distress, and postpartum hemorrhage in the CG were 3, 20, 12, 10, and 12, respectively. The numbers of placenta previa, cesarean section, hyperamniotic fluid, fetal distress and postpartum hemorrhage were 1, 10, 2, 2, and 2, respectively. The frequency of unfavorable pregnancy outcomes was substantially greater in the CG than in the For example, and the number of placenta previa, cesarean section, hyperamniotic fluid, fetal distress, and postpartum hemorrhage decreased by 2, 10, 10, 8, and 10, respectively. The pregnancy outcomes of cesarean section, hyperamniotic fluid, fetal distress and postpartum hemorrhage were significantly different.

### 3.5. Comparison of neonatal status between the 2 groups

Neonatal conditions were divided into neonatal asphyxia, neonatal pneumonia, neonatal hypoxia, neonatal growth retardation and hyperbilirubinemia. Table 4 shows the comparison of the status of newborns born to hypertensive patients during pregnancy.

**Table 4:** Comparison of the status of newborns born to two groups of hypertensive patients during pregnancy.

Compare items	CG	e.g.,	*x^2^*	*P* value
	(n = 40)	(n = 40)		
Neonatal asphyxia	4 (0.1)	11 (0.275)	6.321	<.001
Neonatal pneumonia	0 (0.0)	3 (0.075)	5.324	<.001
Neonatal hypoxia	0 (0.0)	2 (0.05)	6.325	<.001
Neonatal developmental delay	0 (0.0)	4 (0.1)	4.325	<.001
Hyperbilirubinemia	1 (0.025)	6 (0.15)	3.265	<.001
Total	5 (0.125)	26 (0.65)	8.235	<.001

CG = control group.

In Table 4, neonatal asphyxia, neonatal pneumonia, neonatal hypoxia, neonatal growth retardation, neonatal hyperbilirubinemia and total number were 11, 3, 2, 4, 6, and 26 in the CG, while neonatal asphyxia, hyperbilirubinemia and total number were 4, 1 and 5 in the e.g.. Neonatal asphyxia, neonatal pneumonia, neonatal hypoxia, neonatal growth retardation, neonatal hyperbilirubinemia and total number decreased by 7, 3, 2, 4, 5, and 21, respectively. The number of unfavorable neonatal circumstances in the CG was substantially greater (*P* < .05).

### 3.6. Comparison of the 2 groups ratings on the intensity of labor pain

The study used digital pain scoring method to compare the delivery pain of the 2 groups of patients, and Figure 3 depicts the difference in pain ratings between the 2 groups.

**Figure 2.**
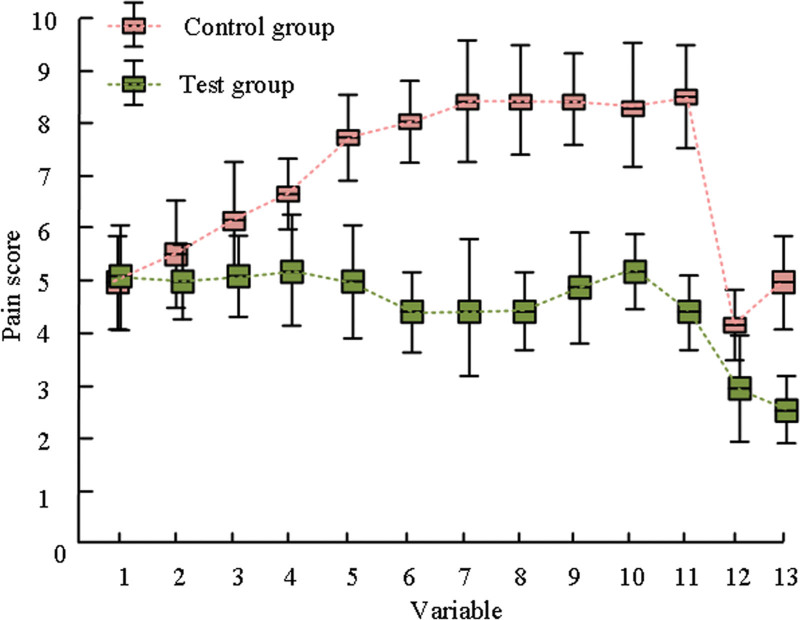
Comparison of pain scores between two groups of patients.

In Figure 2, variables 1 to 10 represent 1 to 10 hours of the first stage of labor, variables 11 and 12 represent the second and third stages of labor, and variable 13 represents 2 hours postpartum. It can be seen that the pain score of the CG is superior to the For example, and the pain change range of the CG is larger than the For example, Although no significant differences in pain after an hour of the first stage of labor were seen across the groups (*P* > .05), there was a difference (*P* < .05) at subsequent points in time.

### 3.7. Comparison of prenatal anxiety between the 2 groups

**Figure 3.**
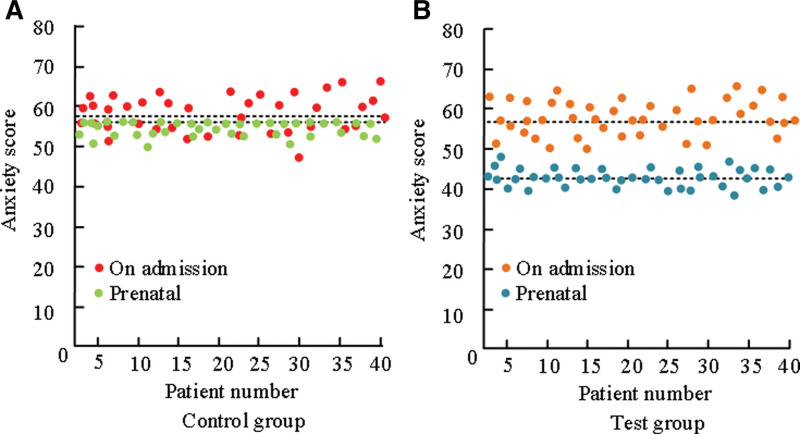
Comparison of prenatal anxiety between two groups of patients.

The degree of anxiety in the study was quantitatively estimated by referring to the clinical anxiety score scale. The comparison of prenatal anxiety between the 2 groups was shown in Figure 3.

In Figure 3, the scores of anxiety degree at admission and prenatal of the CG were 57.32 ± 4.95 and 56.87 ± 4.85, respectively. The scores of anxiety at admission and antenatal were 56.24 ± 5.02 and 43.01 ± 4.23, respectively. The scores of prenatal anxiety in the For example, decreased significantly, while the CG’s prenatal anxiety levels were comparable to those at admission. There were significant differences in prenatal anxiety scores (*P* < .05).

### 3.8. Comparison of blood pressure before and after care between the 2 groups

Calculations were made to determine the diastolic blood pressure and systolic blood pressure. Table 5 demonstrates the comparison of blood pressure before and after care in the 2 groups.

**Table 5:** Comparison of blood pressure between two groups of patients before and after nursing care.

Group	N	Pre care systolic blood pressure (mm Hg)	Post care systolic blood pressure (mm Hg)	Pre care diastolic blood pressure (mm Hg)	Post care diastolic blood pressure (mm Hg)
CG	40	169.12 ± 15.32	135.12 ± 6.25	103.63 ± 10.56	90.45 ± 8.67
e.g.,	40	168.93 ± 15.15	130.93 ± 5.32	103.25 ± 10.27	81.05 ± 7.25
*t*	/	0.0521	3.4586	0.1452	4.6852
*P* value	/	.9586	.0011	.8872	.0001

CG = control group.

In Table 5, CG’s systolic blood pressure before and after care was 169.12 ± 15.32 mm Hg and 135.12 ± 6.25 mm Hg, respectively. The diastolic blood pressure before and after treatment was 103.63 ± 10.56 mm Hg and 90.45 ± 8.67 mm Hg, respectively. The systolic blood pressure before and after the treatment group was 168.93 ± 15.15 mm Hg and 130.93 ± 5.32 mm Hg, respectively. The diastolic blood pressure before and after treatment was 103.25 ± 10.27 mm Hg and 81.05 ± 7.25 mm Hg, respectively. After breastfeeding, the For example, systolic and diastolic blood pressure were much lower than those of the CG, although there was no discernible difference between the 2 groups before nursing(*P* > .05). Both blood pressures were found to be different following breastfeeding (*P* < .05).

### 3.9. Comparison of nursing satisfaction between the 2 groups

**Figure 4.**
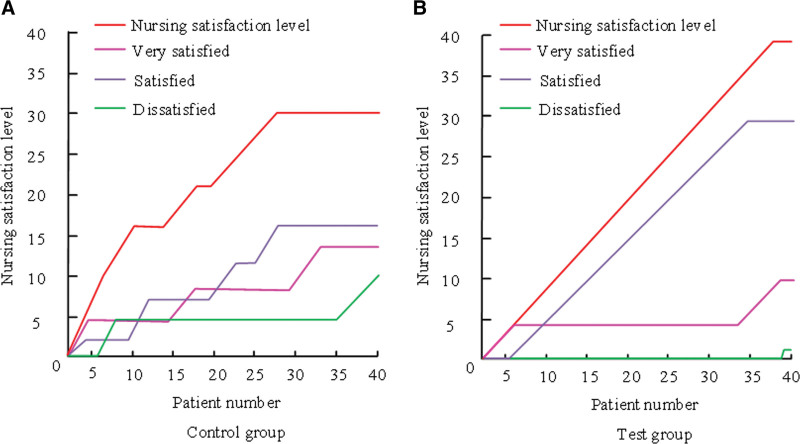
Comparison of prenatal anxiety between two groups of patients.

A questionnaire was applied to quantitatively compare the nursing satisfaction of patients. Nursing satisfaction includes 3 types: very satisfied, satisfied and dissatisfied. The total of very satisfied and satisfied is nursing satisfaction. Figure 4 displays the results of a comparison between the 2 groups in terms of nurse satisfaction.

In Table 4, the very satisfied, satisfied, dissatisfied and nursing satisfaction of the CG were 14, 16, 10, and 26, respectively. Very satisfied, satisfied, dissatisfied and nursing satisfaction of the For example, were 29, 10, 1, and 39, respectively. The For example, level of nursing satisfaction was higher than that of the CG (*P* < .05).

### 3.10. Comparison of the 2 groups quality of life after giving birth

Quality of life conditions include 7 aspects: physical activity, emotional awareness, physical discomfort, mental health, sleep quality, postpartum anxiety or depression, and general health. In this study, the quality-of-life score scale was applied to quantitatively compare the postpartum life quality. The quality of life after giving birth gets better as the number goes up. Table 6 compares the patients postpartum quality.

**Table 6:** Comparison of postpartum quality of life between two groups of patients.

Quality of life situation	CG	EG	*t*	*P* value
Physiological activities	71.6 ± 6.2	78.8 ± 5.4	6.124	.001
Emotional awareness	75.3 ± 5.6	86.9 ± 4.9	10.023	.012
Physical discomfort	76.5 ± 6.4	87.1 ± 6.9	7.465	.023
Mental health	66.5 ± 6.2	77.9 ± 5.6	9.301	.035
Sleep quality	68.5 ± 5.6	80.1 ± 3.5	9.232	0.012
Postpartum anxiety or depression	69.3 ± 3.5	81.2 ± 6.4	8.235	.002
Overall health	70.6 ± 5.3	84.6 ± 6.2	9.063	.003

CG = control group.

In Table 6, the physical activity, emotional awareness, physical discomfort, mental health, sleep quality, postpartum anxiety or depression and overall health scores of the CG were 71.6 ± 6.2, 75.3 ± 5.6, 76.5 ± 6.4, 66.5 ± 6.2, 68.5 ± 5.6, 69.3 ± 3.5, and 70.6 ± 5.3, respectively. The scores of physical activity, emotional awareness, physical discomfort, mental health, sleep quality, postpartum anxiety or depression, and overall health of the EGs were 78.8 ± 5.4, 86.9 ± 4.9, 87.1 ± 6.9, 77.9 ± 5.6, 80.1 ± 3.5, 81.2 ± 6.4, and 84.6 ± 6.2, respectively. The scores of postpartum quality of life in the For example, were far higher than the CG, and the scores of physiological activity, emotional awareness, physical discomfort, mental health, sleep quality, postpartum anxiety or depression, and overall health were statistically significant (*P* < .05).

## 4. Discussion

Hypertensive disease during pregnancy is a complication unique to pregnant women. The incidence of hypertensive disease during pregnancy has been increasing due to the rising life pressure faced by women, which can have severe consequences for both mothers and newborns, including maternal and fetal death.^[16,17]^ Pregnant women with pregnancy-induced hypertension may experience edema and proteinuria after 20 weeks of pregnancy. Mild symptoms include no symptoms or mild dizziness, and severe symptoms include headache or vomiting.^[18,19]^ Relevant studies have shown that maternal tension and anxiety during pregnancy and postpartum will have a great negative impact on it. Especially the prognosis and postpartum life quality, happy and relaxed mood will ensure the basic stability of blood pressure and contribute to the prevention and rehabilitation of pregnant women with hypertension during pregnancy, so health education and nursing are particularly important.^[20,21]^ By changing the nursing process of patients, cluster based care ensures the continuity of patients and supports comprehensive nursing through practice.^[22,23]^ Nursing measures, the strength of opinion evidence and implementation determine the practical effect of cluster based care. Its characteristics are systematic and comprehensive, and it is widely used in clinical nursing of diseases.^[24,25]^ Related studies have demonstrated that cluster care can improve maternal labor pain and postpartum conditions. The IKAP health education theory integrates information, knowledge, belief and behavior organically, based on knowledge, belief, and behavior theory.^[26]^ The process of knowledge acquisition, belief establishment and continuous change of behavior is the step of behavior change.^[27]^ Guided by the principles of patient-centered mental and physical healthcare, IKAP health education constantly promotes health knowledge to patients and their families according to their condition and psychological changes, strengthens their disease cognition and participation in diagnosis and treatment, and positively changes their concepts and living habits. Studies have shown that IKAP health education model can improve patients’ pregnancy outcomes and newborn health conditions.^[28]^

In this study, patients with pregnancy-induced hypertension were chosen as the study objects to analyze the application effect of IKAP health education combined with cluster-based care. There were no notable variations in age, prepregnancy BIM and education level (*P* > .05). The diet control ratio of For example, and CG was 97.5% and 70%, respectively. The proportion of appropriate exercise was 97.5% and 67.5% respectively. The proportion of regular blood pressure measurement was 95% and 50% respectively (*P* < .05), indicating that IKAP health education combined with diet control, appropriate exercise and regular blood pressure measurement of cluster care had a more significant effect than that of cluster care alone. In the CG, the measurements of amniotic fluid index, BMI, pregnancy weight gain, and blood pressure were 125.81 ± 30.21 mm, 21.64 ± 1.82 kg/m^2^, 17.81 ± 2.54 kg and 135.12 ± 6.25 mm Hg, respectively. The measurements of amniotic fluid index, BMI, pregnancy weight gain, and blood pressure were 109.83 ± 28.72 mm, 20.51 ± 1.62 kg/m^2^, 16.59 ± 2.84 kg, and 130.34 ± 5.32 mm Hg, respectively. There were statistically significant variations in pregnancy markers, including amniotic fluid index, BMI, weight gain during pregnancy, and blood pressure and hypertension (*P* < 0.05). The For example, pregnancy index greatly outperformed the CG’s, indicating that IKAP health education combined with cluster-based care had more obvious control effect. Prenatal anxiety levels showed statistically significant variations (*P* < 0.05). The anxiety degree of the For example, was reduced more than the CG, indicating that the effect of IKAP health education combined with cluster care on the anxiety relief of patients was better than that of cluster care alone. In contrast to the For example, the incidence of unfavorable pregnancy outcomes was considerably greater in the CG. The results of the pregnancies differed statistically significantly (*P* < .05), indicating that IKAP health education combined with cluster-based care can help reduce the probability of adverse pregnancy. Compared with the CG, neonatal asphyxia, neonatal pneumonia, neonatal hypoxia, neonatal growth retardation, neonatal hyperbilirubinemia, and total number in the For example, were reduced by 7, 3, 2, 4, 5, and 21, respectively, indicating that IKAP health education combined with cluster care can reduce the frequency of neonatal adverse conditions. The systolic blood pressure of the CG before and after nursing was 169.12 ± 15.32 mm Hg and 135.12 ± 6.25 mm Hg, respectively. The diastolic blood pressure before and after treatment was 103.63 ± 10.56 mm Hg and 90.45 ± 8.67 mm Hg, respectively. The systolic blood pressure before and after the treatment group was 168.93 ± 15.15 mm Hg and 130.93 ± 5.32 mm Hg, respectively. The diastolic blood pressure before and after treatment was 103.25 ± 10.27 mm Hg and 81.05 ± 7.25 mm Hg, respectively. Both systolic and diastolic blood pressure were considerably lower in the For example, after breastfeeding compared to the CG (*P* > 0.05). The difference of systolic and diastolic blood pressure after nursing was statistically significant (*P* < .05), indicating that IKAP health education combined with cluster-based care had more effective blood pressure management. The scores of physical activity, emotional awareness, physical discomfort, mental health, sleep quality, postpartum anxiety or depression, and overall health of the EGs were 78.8 ± 5.4, 86.9 ± 4.9, 87.1 ± 6.9, 77.9 ± 5.6, 80.1 ± 3.5, 81.2 ± 6.4, and 84.6 ± 6.2, respectively. Compared to the CG, the For example, had considerably better postpartum quality of life ratings. Significant variations in the postpartum quality of life (physiological activity, emotional awareness, physical discomfort, mental health, sleep quality, postpartum anxiety) or depression, and overall health scores between the 2 groups were found statistically (*P* < 0.05).

In summary, IKAP health education combined with cluster care can further improve postpartum patients physiological and psychological states. However, there are still areas for improvement in the research. Further improvements can be made by increasing the sample size to enhance the scientific validity and accuracy of the study findings.

## Author contributions

**Data curation:** Jiao Wen.

**Formal analysis:** Jiao Wen.

**Investigation:** Xiuping Liu.

**Methodology:** Jiao Wen.

**Supervision:** Xiuping Liu.

**Writing – original draft:** Jiao Wen.

**Writing – review & editing:** Jiao Wen, Xiuping Liu.
